# Cortical Temperature Change: A Tool for Modulating Brain States?^1^,^2^

**DOI:** 10.1523/ENEURO.0096-16.2016

**Published:** 2016-06-22

**Authors:** Miriam Schwalm, Curtis Easton

**Affiliations:** 1 Focus Program Translational Neuroscience (FTN), and Institute for Microscopic Anatomy and Neurobiology, Johannes Gutenberg-University Mainz, 55128 Mainz, Germany; 2 GRADE Brain, Goethe Graduate Academy, Goethe University, 60325 Frankfurt, Germany

**Keywords:** brain states, epilepsy, neuromodulation, sleep, slow-wave rhythms, waking state

## Significance Statement

This commentary describes important findings of the article published by Sheroziya and Timofeev in The Journal of Neuroscience in 2015. The authors use moderate cortical temperature change, local cooling or heating of somatosensory cortex, to modulate excitable states of the brain. These changes, under physiological conditions, result from neuromodulation, as well as other network effects. They report that cooling disrupts thalamocortical slow oscillations and induces an activated cortical state, while mild heating has the opposite effect and increases slow-wave rhythmicity. We evaluate these findings regarding their utility for inducing and investigating cortical state fluctuations, compare the results to physiologically occurring state changes, and put them into perspective with other discoveries in the field.

## 

Periods of rhythmic slow-wave activity during physiological slow-wave sleep or induced by anesthesia are characterized by a waxing and waning of spontaneous neuronal firing coordinated between cortex and thalamus. This activity is generated in the cortex but influences neuronal excitability and stimulus–response properties of neuronal networks throughout the brain (Steriade et al., 1993; Stroh et al., 2013; McGinley et al., 2015b). The corresponding low-frequency component of field potential recordings reflects alternating active states, in which cells are depolarized and synaptic activity is high, and silent states with hyperpolarized membrane potentials and low synaptic activity (Steriade et al., 2001; Timofeev et al., 2001). In contrast, waking is generally associated with continuous depolarization of cortical neurons, resulting in persistent activity (Destexhe et al., 2007; Sheroziya and Timofeev, 2015) and suppression of silent states (Steriade et al., 2001; McGinley et al., 2015b). In their recent study, Sheroziya and Timofeev (2015) demonstrated that moderate cortical cooling (to 29–31°C) of lightly ketamine/xylazin (ket/xyl) anesthetized or non-anesthetized mice reversibly diminished silent states and induced a persistent active state of the cortex. Mild heating (to 39–40°C), in contrast, increased rhythmicity of slow waves. Under deep ket/xyl anesthesia, cortical cooling disrupted slow waves and promoted bursts of activity correlated with thalamic firing. Local cooling of somatosensory cortex was shown to be sufficient to induce a shift from slow-wave to wide-spread persistent cortical activity, extending to the thalamus as well as the contralateral hemisphere. These results suggest that cortical temperature change can be used as a bidirectional and reversible tool for investigating global brain state fluctuations, and provide evidence that the thalamocortical network rapidly reacts upon local depolarization of a small neuronal population with wide-spread shifts of brain state.

An effect of cortical cooling on thalamocortical slow waves has previously been reported (Kalmbach and Waters, 2012). These authors examined consequences of temperature loss in cortical areas underneath a cranial window preparation and showed that a decline of surface temperature to 28°C leads to depolarization and reduction of silent states in whole-cell recordings of layer 2/3 pyramidal neurons. They compared these datasets to recordings performed under constant perfusion of warmed solution over the craniotomy, resulting in 36–38°C of cortical temperature, similar to control conditions of Sheroziya and Timofeev (2015). Kalmbach and Waters (2012) observed a reduction of slow-wave synchrony during cooling, characterized by diminished silent and prolonged active states. As they used relatively high isoflurane anesthesia, these results are comparable with the results of Sheroziya and Timofeev (2015) acquired under deeply anesthetized conditions. Sheroziya and Timofeev (2015) examined cooling-induced active states also in non-anesthetized animals where thalamic and contralateral recordings demonstrated a widespread effect of cooling far beyond cortical layer 2/3. Cooling prevented the generation of slow waves, and the authors state that it appeared to elicit a sensory experience that excited the animals, although this effect was not extensively described. Thus, the work of Sheroziya and Timofeev (2015) goes beyond previous studies about the effects of temperature on cortical activity, showing that temperature change can be used to reversibly induce global cortical state fluctuations in awake or physiologically sleeping animals without directly influencing or manipulating neuromodulation.

Although the exact conditions leading to spontaneous brain state shifts remain unknown, several pathways have been described in this process, and the effects of cooling may overlap mechanistically with these pathways. In awake but restful animals, slow waves may be observed and are suppressed by the initiation of whisking or locomotion (Crochet and Petersen, 2006) or firing of thalamic neurons (Poulet et al., 2012), which speaks for underlying neuromodulatory influences (Lee and Dan, 2012). Recently, state shifts in cortical networks have been directly related to neuromodulatory pathways, especially the cholinergic (Eggermann et al. 2014; McGinley et al., 2015a) and the noradrenergic (McGinley et al., 2015a) system. For example, selective optogenetic stimulation of cholinergic axons in the cortex leads to desynchronization of cortical local field potentials (Kalmbach et al., 2012). Sheroziya and Timofeev (2015) claim that, similar to neuromodulator release, moderate temperature decrease leads to depolarization of neurons by a partial closure of potassium channels and reduction of synaptic release in a manner similar to, but independent from neuromodulation. As mentioned by the authors, reduced activity of potassium channels directly leads to depolarization of neurons and thereby could induce a desynchronized active state. The mentioned reduction of synaptic release by cooling may contribute to this effect through local disinhibitory circuit mechanisms (McGinley et al., 2015b). Local reduction of synaptic release in the cortex, as it is the case during cooling, may favor excitation by activating disinhibitory pathways, which can increase the level of excitability of cortical pyramidal cells (Lee et al., 2013; Fu et al., 2014; McGinley et al., 2015b) and thereby contribute to the network shift toward an active state. Cooling itself was shown to disrupt inhibitory circuits at least in hippocampal slices (Javedan et al., 2002), which could further contribute to this effect. Moreover, McGinley et al. (2015b) propose several mechanisms for transitioning between slow-wave and active brain states in the awake animal which may also be relevant to the cooling-induced cortical activation. Analogous to acetylcholine, cooling may modulate cortical activity through subcellular effects. For example, the authors show a strong reaction to cooling in the ket/xyl anesthetized animals that might be explained by the actions of xylazin as an α2 adrenoreceptor agonist that depolarizes layer 5 pyramidal cells (McCormick, 1992). This may disrupt oscillatory activity in this cortical layer which is critical to slow-wave generation (Sanchez-Vives and McCormick, 2000), and thus may contribute to slow-wave suppression during cooling.

McGinley et al. (2015b) further introduce two models of transitions between states in physiological conditions which may be relevant for evaluating the temperature induced state shifts. First, they suggest a sigmoidal relationship between cellular membrane potential and arousal state, wherein the transition from low to medium arousal causes cells to exhibit depolarized membrane potentials, and the shift to high arousal causes further depolarization of the network. In contrast, they present a U-shaped model, where moderate arousal suppresses slow-wave oscillations and hyperpolarizes neurons, and only further arousal causes neuronal depolarization. Both models predict depolarized membrane potentials due to the appearance of gamma activity at high arousal levels. Synchronized gamma (>40Hz; Singer and Gray, 1995) is characteristic of persistent, global brain activity, including representations of specific stimuli. Gamma activity increases in the cortical EEG (Steriade et al., 2001) after a transition to waking. In awake animals, spontaneous or induced rises of arousal level are accompanied by a monotonic increase in gamma band synchronization (Lima et al., 2011; McGinley et al., 2015a). The depolarization of cortical neurons (Kalmbach and Waters, 2012) and the increase of gamma activity (Sheroziya and Timofeev, 2015) upon cooling under deeper anesthesia mimic initial arousal from slow-wave state and support the sigmoidal model. Depolarization is explained by the increase of spontaneous active states with reduction of silent states, leading to more depolarized membrane potentials because of the dominating high-frequency components. However, rather hyperpolarized membrane potentials and decreased gamma activity during light anesthesia and cortical cooling (Sheroziya and Timofeev, 2015) supports the U-shaped model. Here, suppression of slow-wave activity is accompanied by reduced power in the gamma frequencies because bursting activity during slow waves no longer occurs. At present, both models are supported by cortical recordings under various physiological conditions, and may be related to differential impact of neuromodulatory release (McGinley et al. 2015b). The effects of cortical cooling fit the predictions of the two models, which supports the notion that the cooling-induced state-changes recapitulate physiological neuronal network dynamics. This is also in agreement with the idea that each of the models may be more applicable depending on experimental conditions, physiological factors, such as the type of arousal leading to state changes, or the initial state of wakefulness of the animal.

However, the question remains whether a cooling-induced state leads to neuronal response properties comparable to those of physiological active states of the cortex. Although obtained results share characteristics of spontaneous physiological state shifts, other widespread effects due to cooling cannot be completely excluded. For example temperature change has been shown to affect brain pH (Schuchmann et al., 2002, 2006), and could have effects on cerebral blood flow with accompanied compensational mechanisms. In addition to the effects on potassium channels and transmitter release discussed by the authors, temperature change will also exhibit strong effects on mitochondrial activity, with cooling resulting in reduced ATP synthesis and thereby decreased activity of sodium–potassium pumps. The resulting depolarization will inevitably affect glutamate and GABA uptake, which may impede a direct comparison to a physiologically active state. Additionally, it is not clear whether the shown cooling effects simply result from slowed ion channel kinetics and reduced network excitability so that a highly active state, such as the synchronous neuronal activity during slow oscillations, can no longer be supported. The network might default to a desynchronized state where individual neurons may still fire rapidly, but overall neural excitability is reduced in a way, which does not replicate a physiologically awake state. This concept is supported by Sheroziya and Timofeev’s (2015) Figure 3, which shows shorter interspike intervals for neurons in the synchronous state versus the cooled state. Such a breakdown of network synchrony locally in the cooled cortex may propagate to other cortical regions due to effects of local desynchronization on the thalamocortical network. This hypothesis could be tested by applying sensory stimulation during cooling conditions and comparing neuronal responses to those observed in awake animals; for example, measuring firing rates in somatosensory cortex upon whisker stimulation in cooled versus control conditions. If the desynchronizing effect of cooling is simply due to lower neuronal excitability, then this would be reflected in a reduced response of cortical neurons to sensory stimulation in the cooled versus awake condition.

Finally, although therapeutic hypothermia is often used to prevent or control seizures, it is argued that moderate cortical cooling has a light epileptogenic effect (Sheroziya and Timofeev, 2015). This is due to similarities observed between cooling-induced cortical discharges and cortical activity during spike-wave absence seizures, and contrasts with another study showing that moderate, focal cooling can prevent seizures in a rodent injury model of epilepsy (D’Ambrosio et al., 2013). It was debated that the effects of hypothermia on metabolism may explain neuroprotective actions, and that the changes to cellular membrane properties actually counter the neuroprotective effects of cooling with regard to epilepsy. However to fully understand the relevance of cooling-induced changes on cellular activity to the treatment of epilepsy, it is necessary to consider a broader range of epileptic conditions. Overall, the described desynchronizing effect of hypothermia on neuronal activity may provide the best neuroprotective element for epilepsy treatment. Brain state plays an important role in seizure generation, as epilepsy is associated with sleep disturbance (St Louis, 2011; Kalume et al., 2015), and many seizures are more likely to occur or generalize during slow-wave sleep. For example, Herman et al., (2001) compared the initiation and generalization of partial, frontal, and temporal lobe seizures and concluded that non-REM sleep was most conducive to seizure generation and that the hypersynchrony of this sleep state facilitates initialization and generalization of partial seizures. This is consistent with a therapeutic effect of cortical cooling in epilepsy, based on the interruption of hypersynchrony associated with slow waves. Additionally, the enhancement of slow-wave activity with cortical heating is consistent with hypersynchrony as a trigger for seizures because of the tendency for seizures to be generated with fever, although this phenomenon has also been linked to respiratory alkalosis and changes to brain pH with high body temperatures (Schuchmann et al., 2006).

In conclusion, we find that the authors provide intriguing evidence in support of temperature changes as a tool to modulate brain states. Although further studies need to evaluate whether the cooled state represents persistent population activity comparable to physiological active states of the thalamocortical network, the present study adds valuable information to current knowledge about the nature and mechanisms of cooling-induced desynchronization. Additionally it provides evidence that mild heating may be used to synchronize brain networks. The implications for these findings could be far reaching, with applications in studies of both the mechanisms of brain state shifts and the use of temperature in the treatment of diverse diseases from epilepsy to sleep disturbances.

## Synthesis

The decision was a result of the Reviewing Editor Uwe Heinemann and the peer reviewers coming together and discussing their recommendations until a consensus was reached. A fact-based synthesis statement explaining their decision and outlining what is needed to prepare a revision is listed below. The following reviewers agreed to reveal their identity: Yuri Zilberter, Martin Holtkamp

The manuscript provides a useful review of the important article by Sheroziya&amp;Timofeev (J Neurocsi, 35,2015), highlighting the most significant aspects of this research. The reviewers find the commentary very useful. There are only minor changes required.

1. Lowering temperature or increasing temperature have strong effects of mitochondrial activity resulting in case of cooling in reduced ATP synthesis and thereby decreased activity of the sodium potassium pump. The resulting epolarisation will also affect glutamate and GABA uptake.Thus the "cooled" state can not be compared to the physiologically active one. Not only K channels and transmitter release are affected. Thus, cooling is a nice tool for switching between brain states, but not the physiological states indeed. I guess this aspect is not elucidated sufficiently by Sheroziya&amp;Timofeev.

2. L35: please clarify, what do you mean by "critical evidence" specifically.

3. L144: we agree completely, acute de-synchronization most likely provides the cooling anti-seizure effect. This is not mentioned by Sheroziya&amp;Timofeev and their reference to (Erecinska et al., 2003) is not appropriate for this specific case. But see a study by Schuchmann et al on effects of coling on different types of epileptiform activity.
